# A revision of the genus *Littorina* (Mollusca: Gastropoda) in Korea

**DOI:** 10.1080/19768354.2018.1545697

**Published:** 2018-11-14

**Authors:** Yucheol Lee, Youngjae Choe, Elizabeth M. A. Kern, Yeongheon Shin, Taeho Kim, Joong-Ki Park

**Affiliations:** a Department of Biological Sciences, Sungkyunkwan University, Suwon, Korea; b Division of EcoScience, Ewha Womans University, Seoul, Korea

**Keywords:** Korea, *Littorina*, Littorinidae, Radula, mtDNA *cox1*

## Abstract

*Littorina* Férussac, 1822 is an abundant genus of small gastropods found in the upper littoral zone of rocky seashores worldwide. Although ecologically important, shell-based species identification in this genus is challenging due to phenotypic variation in shell morphology and lack of diagnostic characters among morphologically similar species. In this study, we revised the taxonomy of Korean *Littorina* species using morphological characters (shell and radula) and *cox1* mitochondrial DNA sequences for three Korean species: *L. brevicula*, *L. sitkana,* and *L. horikawai*. Results suggest that *L. sitkana* was erroneously reported as *L. kasatka* in a previous study. A new record for *Littorina horikawai* (Matsubayashi & Habe in Habe, 1979), previously unknown from Korea, is described, which can be distinguished from *L. sitkana* by the presence of alternating white and brown spiral ribs on each whorl. Comparison of the mtDNA *cox1* gene sequences shows very low intraspecific variation even between geographically distant populations. A phylogenetic tree supports a close relationship between *L. horikawai* and *L. sitkana*, consistent with earlier phylogenetic studies.

## Introduction

The family Littorinidae include nearly 180 species from 13 genera worldwide, including 11 species from 6 genera in Korea (Lee and Min 2002; Min et al. 2004; Lee and Kil 2013; this study). Within this family, members of the genus *Littorina* Férussac, 1822 (commonly known as periwinkles) are among the most abundant gastropods in the upper littoral zones of rocky seashores throughout the temperate region of the northern hemisphere. They comprise 18 species worldwide (Reid et al. 1996; 2012) and have been used as models in evolutionary ecology (Rolan-Alverez et al. 2015) and as pollution biomonitors (Kang et al. 2000; Noventa and Pavoni 2011). Some members of this group show considerable intraspecific variation in shell color and sculpture, making identification challenging by shells alone (Struhsaker 1968; Reid et al. 1996; Johannesson 2016).

Previous records of *Littorina* species in Korea have occasionally contained taxonomic errors, which calls for species descriptions to be revisited and previous diagnoses reexamined. To date, three *Littorina* species have been recorded from Korean waters: *L. brevicula* (Philippi 1844), *L. sitkana* Philippi, 1846, and *L. kasatka* Reid, Zaslavskaya & Sergievsky, 1991 (Min et al. 2004; Lee and Kil 2013). Among these species, *L. sitkana* and *L. kasatka* in particular are difficult to tell apart by shell morphology alone, and have overlapping ranges (Reid 1996). Here we revise Korean *Littorina* species using additional sampling and identification efforts, provide detailed descriptions of shell and radula morphology, and present a new record of *Littorina horikawai* with a description of shell characters. Mitochondrial cytochrome *c* oxidase subunit 1 (*cox1*) gene sequences were determined for *L. brevicula*, *L. sitkana* and *L. horikawai* and used for molecular identification.

## Materials and methods

### Sample collection and species identification

Specimens of *Littorina* were collected from the intertidal zone of South Korea. *L. horikawai* (a new record from Korean waters) were collected from the rocky shore of Jeju Island, Korea (Table 1). All samples were preserved in 95% ethanol. For species identification and descriptions, shell morphology was examined using a stereoscopic microscope (Leica M205C, Wetzlar, Germany). Voucher specimens of the examined materials were deposited in the Marine Mollusk Resource Bank of Korea (MMRBK). Species identification of Korean *Littorina* species was based on morphological descriptions by Reid et al. (1996) and confirmed by molecular identification using mitochondrial *cox1* sequences.

**Table 1.** Samples and GenBank accession numbers used for phylogenetic analysis in this study.

Species	Range	Collection location latitude/longitude	GenBank accession number
*Littorina brevicula* 1^a^	Korea	Uido-ri 34°37′06.76″ / 125°51'16.46″	KY752070
*Littorina brevicula* 2^a^	Korea	Modong-ri 34°11′57.90″ / 126°46′06.79″	KY752071
*Littorina brevicula*	Korea	–	KU977417
*Littorina mandshurica*	Japan	–	HE590838
*Littorina littorea*	Canada	–	KF644330
*Littorina squalida*	Japan	–	HE590843
*Littorina kasatka*	Japan	–	HE590837
*Littorina plena*	USA	–	AJ622948
*Littorina scutulata*	Canada	–	KX069595
*Littorina aleutica*	Russia	–	HE590831
*Littorina natica*	Russia	–	HE590839
*Littorina compressa*	France	–	HE590834
*Littorina saxatilis*	Canada	–	KF644164
*Littorina arcana*	France	–	HE590832
*Littorina fabalis*	United Kingdom	–	HE590835
*Littorina obtusata*	Canada	–	KF644236
*Littorina subrotundata*	Russia	–	HE590844
*Littorina horikawai*^b^	Korea	Samyang-dong 33°31′38.99″ / 126°35′13.96″	KY752072
*Littorina horikawai*	Japan	–	HE590836
*Littorina sitkana*^a^	Korea	Ayajin-ri 38°16′13.35″ / 128°33′28.75″	KY752073
*Littorina sitkana*	Canada	–	KF644171
*Littorina keenae*	USA	–	AJ488629
*Echinolittorina radiata*	Japan	–	AJ623040

^a^Determined in this study.

^b^New record in Korea.

### Scanning electron microscopy (SEM) of the radula

The radula ribbon was extracted from the buccal mass of each dissected animal and cleaned in a 10% KOH solution. After being rinsed with distilled water several times, radula samples were dried using a Hitachi HCP-2 critical point drier, mounted on copper/nickel tape attached to a SEM stub, and gold-palladium coated using an Eiko IB-3 sputtercoater. Radulae were observed using a Zeiss Ultra Plus SEM at 15 kV under high-vacuum conditions.

### Molecular techniques and phylogenetic analysis

Genomic DNA was extracted from foot tissue using an E.Z.N.A. mollusc DNA kit (OMEGA Omega Bio-tek, Norcross, USA) following the manufacturer’s instructions. Using the LCO1490 and HCO2198 primer set (Folmer et al. 1994), the mitochondrial *cox1* fragment was PCR-amplified in 50 μl of TaKaRa Ex *Taq* PCR mixture containing 2 μl of template DNA, 34.75 μl of D.W., 5 μl of 10x Ex *Taq* Buffer, 2 μl of primer set, and 0.25 μl of TaKaRa Ex *Taq* under the following conditions: 1 cycle of denaturation at 95°C for 2 min followed by 40 cycles of denaturation at 94°C for 30 s, annealing at 45°C for 30 s, elongation at 72°C for 1 min, and a final extension at 72°C for 10 min. The PCR-amplified target fragment was purified using a Qiaquick gel extraction kit (Qiagen Valencia, USA) and sequenced using an ABI PRISM 3700 DNA analyzer (Applied Biosystems, Foster City, USA). *Cox1* sequences of *L. brevicula, L. sitkana* and *L. horikawai* were deposited in GenBank (accession numbers in Table 1). The *cox1* sequences from the three Korean *Littorina* species and the homologous *cox1* sequences of 18 *Littorina* species from GenBank (Table 1) were used for phylogenetic analysis with *Echinolittorina radiata* as an outgroup, using maximum likelihood and Bayesian methods.

## Results

### Morphology

**Figure 1.**
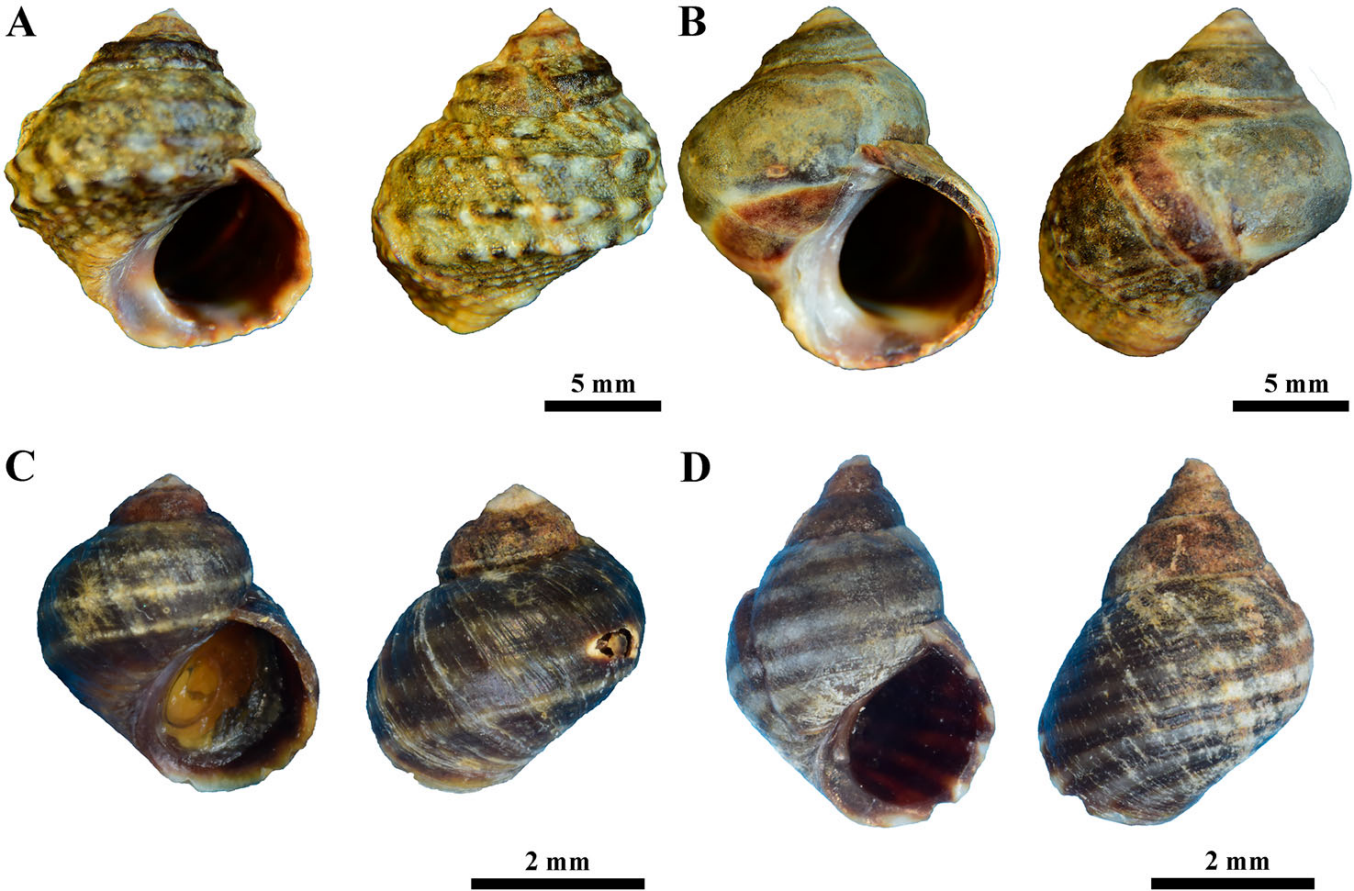
Shells of the three Korean Littorina species. Left. Ventral view; Right. Dorsal view. A = L. brevicula (Philippi, 1844) from Uido-ri. B = L. brevicula (Philippi, 1844) from Modong-ri. C = L. sitkana Philippi, 1846. D = L. horikawai Matsubayashi & Habe in Habe, 1979.

**Figure 2.**
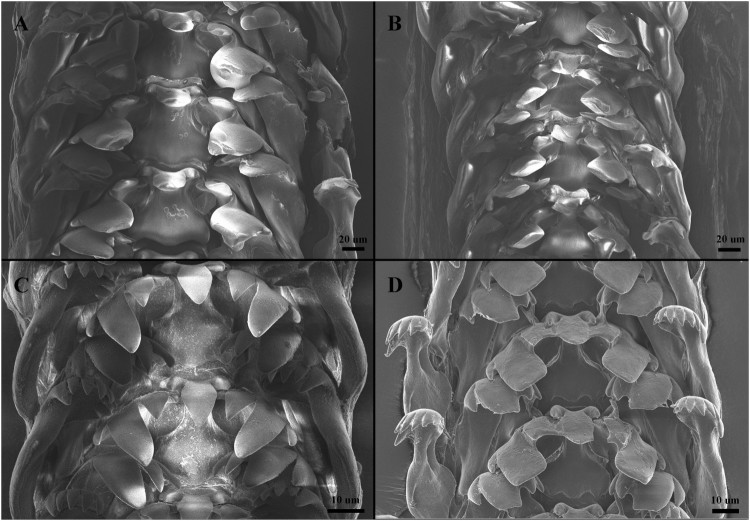
SEM images of radulae for the three Korean Littorina species. A = L. brevicula (Philippi, 1844) from Uido-ri. B = L. brevicula (Philippi, 1844) from Modong-ri. C = L. sitkana Philippi, 1846. D = L. horikawai Matsubayashi & Habe in Habe, 1979.

Shell and radula morphology of the three Korean *Littorina* species are illustrated in Figures 1 and 2.

Familly Littorinidae Children, 1834

Genus *Littorina* Férussac, 1822

***Littorina brevicula* (Philippi, 1844)   Fig. 1A, 1B (shell); Fig. 2A, 2B (radula)**

*Turbo brevicula* Philippi, 1844: 166.

*Litorina brevicula*: Philippi, 1847: 161–162, pl. 3, fig 10, fig 43.

*Littorina brevicula*: Reeve, 1857: sp. 51, pl. 10, fig 51a, b; Reid, 1996: 127–138, figs 43–47.

*Littorina sitkana*: Min et al., 2004: 132–133, figs 234–1, 234–2.

**Materials examined.** 3 individuals, Jeollnam-do, Shinan-gun, Docho-myeon, Uido-ri, Korea, 24 September 2008; 2 individuals, Jeollanam-do, Wando-gun, Cheongsan-myeon, Modong-ri, Korea, 30th October 2012.

**Measurements.** Height 18–19 mm; width 15–16 mm

**Shell morphology:** Shell turbinate in shape. Each whorl and apex eroded. Body whorl occupies more than one-half of the shell length, with 3–5 strong spiral ribs or weak spiral ribs on body whorl; color dark brown; ribs usually with light brown and white spots or white banded pattern. Suture shallow, but each whorl distinct. Aperture wide-oval in shape, color brown with white basal band. Outer lip irregular, light brown. Basal lip thick; interior of shell polished and dark brown with white bands on its surface (a reflection of the external surface banding pattern).

**Radula:** Central tooth maple-leaf shaped when viewed from front; Central cusp large and elongated, pointed, with one small outer denticle on each side. Lateral tooth with 4 cusps; the third from inside being largest, with a pointed tip; the other cusps triangular and pointed. Outer marginal tooth ladle shaped: 5–7 pointed cusps; narrow neck.

**Distribution in Korea:** very common throughout the coastal areas

***Littorina sitkana*Philippi, 1846   Fig. 1C (shell);Fig. 2C (radula)**

*Littorina sitkana* Philippi, 1846: 140; Reid, 1996:146–162, figs 54–56.

*Littorina kasatka*: Lee & Kil, 2013: 87–89, fig 1.

**Materials examined.** 4 individuals, Gangwon-do, Goseong-gun, Toseong-myeon, Ayajin-ri, Korea, 25th May 2011.

**Measurements.** Height 4–4.5 mm; width 3–3.5 mm

**Shell morphology:** Shell small-oval in shape; surface smooth or sculptured with spiral grooves. Each whorl and apex eroded. Body whorl occupies more than one-half of the shell length; weak spiral ribs, with 2–3 yellowish white bands on body whorl. Suture shallow, but each whorl distinct. Aperture oval in shape, dark brown in color. Outer lip irregular, thin and smooth, yellow to white in color. Basal lip thick, and interior of shell polished and dark brown. Shell sculpture and color pattern areknown from previous work to be highly variable by geographic origin (see Reid 1996 for details). The shell characters and measurements described in this study were based purely on a few samples collected from eastern coast of Korea.

**Radula:** Central tooth maple-leaf shaped when viewed from front; central cusp large and elongate-pointed; one small outer denticle on each side. Lateral tooth with 4 cusps; the third-from-inside cusp large and pointed, the other cusps triangular and pointed. Outer marginal tooth ladle shaped: 8 pointed cusps; narrow neck. The radula formula and shape of the *Littorina* species are very similar, and comparison of radula characters provides little information for discrimination between these species.

***Littorina horikawai* Matsubayashi & Habe inHabe, 1979  Fig. 1D (shell);Fig. 2D (radula)**

*Littorina horikawai* Matsubayashi & Habe, in Habe, 1979: 2–3; Reid, 1996: 162–168, figs 59–61. Okutani, 2000: 142–143. Pl. 71, fig. 32.

**Materials examined.** 21 individuals, Jeju Island, Jeju-si, Samyang-dong, Korea, 2nd October 2014.

**Measurement.** Height 4–6 mm; width 3–4 mm

**Shell morphology:** Shell small-oval in shape. Each whorl and apex eroded. Body whorl occupies more than two-thirds of the shell length; weak spiral ribs, dark brown and white bands arranged alternatively in each whorl, extending to outer lip in the body whorl. Suture shallow, but each whorl distinct. Aperture round-oval in shape. Outer lip curved slightly outward. Basal lip thick and interior of shell polished and dark purple, with alternating white and dark brown bands on its surface (a mirror image of the external banding pattern). Outer lip oval.

**Radula:** Central tooth maple-leaf shaped; major cusps large and elongate-pointed; one small outer denticle on each side. Lateral tooth with 4 cusps; the third-from-inside cusp large and rectangular or pointed, the other cusps triangular and pointed. Outer marginal tooth ladle shaped; 8 pointed cusps; narrow neck.

### Molecular identification of Korean *Littorina* species

**Figure 3.**
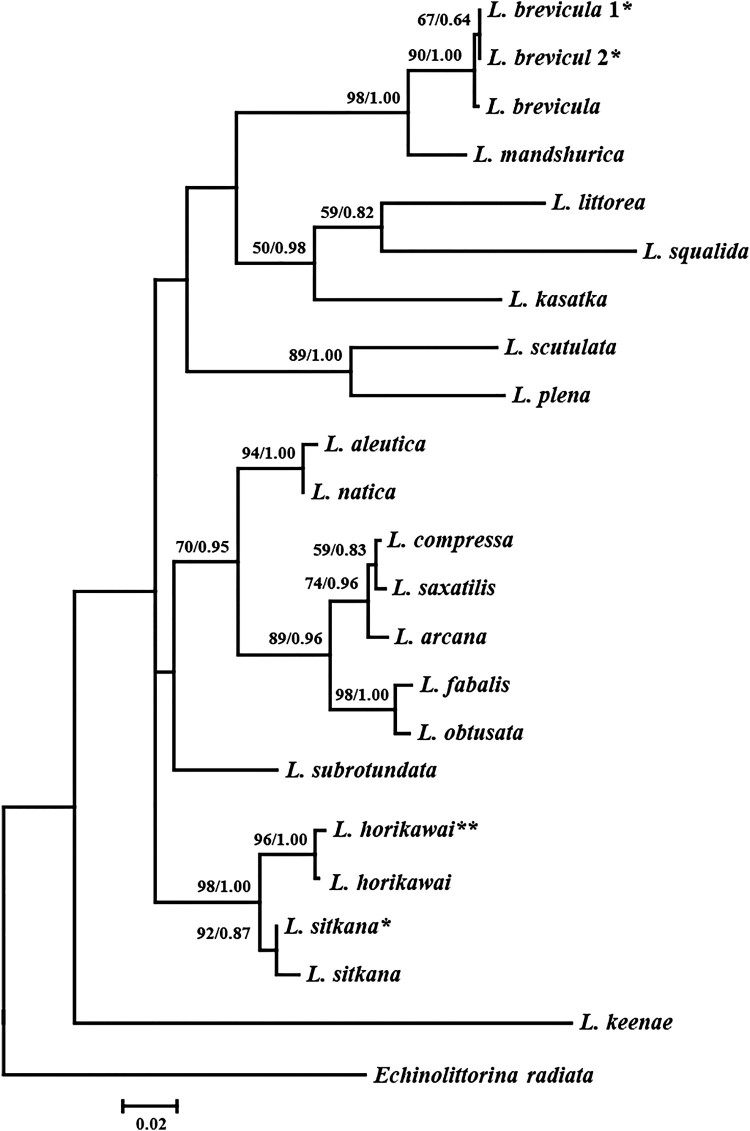
Phylogenetic tree showing the relationships among Littorina species inferred from partial mitochondrial cox1 sequences using maximum-likelihood and Bayesian methods. Numbers above branches are branch support values (bootstrap values/posterior probability values). *: determined in this study, **: new record in Korea.

Species identification of this genus based on shell morphology alone is often challenging due to ecotypic shell variation, as mentioned in previous studies (see Reid 1996 for more details). In order to confirm species identification of the three sampled Korean *Littorina* species, mtDNA *cox1* gene fragment sequences were determined and compared with homologous gene sequences of 18 *Littorina* species on GenBank. Intraspecific sequence divergence of the three Korean *Littorina* species was very low (less than 1%), and phylogenetic analyses showed that *cox1* sequences of the same species clustered together (Table 1, Figure 3): the *cox1* sequences of the two Korean *L. brevicula* individuals collected from Modong-ri and Uido-ri were identical to each other and clustered with the *L. brevicula* sequences on GenBank (differing from each other by a maximum of 2 bp [KU977417]). Our Korean *L. sitkana cox1* sequences differed from the GenBank *L. sitkana* sequences (KF643536, KF644171: British Columbia, Canada) only by 4 bp. Considering the geographic separation between the northwestern Pacific (NWP) and northeastern Pacific (NEP) populations, it is interesting to note the very low sequence divergence (at most 0.5%) between Korean and Canadian isolates of *L. sitkana*. This result supports the hypothesis of recent, eastward trans-pacific migration after the last glacial period (Azuma et al. 2017). The *L. horikawai cox1* sequence from this study also clustered with the *L. horikawai* sequence from GenBank (differing by 3 bp from HE590836: Hirado Is., Nagasaki, Japan).

## Discussion

*Littorina* species are difficult to distinguish based on shell morphology alone. Species in this genus have a small-sized body and the shell surface is often abraded. Moreover, ecotypic variation in shell sculpture in this genus often leads to identification errors. In such cases, utilization of molecular data can be very useful for confirming species identification. We identified three Korean *Littorina* species (*L. brevicula*, *L. sitkana,* and *L. horikawai*) based on morphology, and confirmed their identification using phylogenetic analysis based on mitochondrial *cox1* gene sequences. The phylogenetic analysis with 18 *Littorina* species showed that each of the three Korean species clustered with the respective haplotypes of their matching species from GenBank, supporting our morphology-based identification (Figure 3).

Based on morphological examinations of shell and radula morphology as well as molecular evidence, we conclude that a previous record identified as *L. kasatka* by Lee and Kil (2013) is likely to be a misidentification of *L. sitkana*. These two species can be often confused due to their nearly identical shell characters and broad within-species variation (Reid 1996). We found that the mtDNA *cox1* sequence obtained from the sample that Lee and Kil (2013) used for their morphology-based identification of *L. kasatka* was nearly identical (up to 4 bp) to the *cox1* sequence of *L. sitkana* on GenBank. Further morphological re-examination of Lee and Kil (2013)’s specimens (identified by them as *L. kasatka*) corresponds to the original description of the shell morphology of *L. sitkana* (Philippi 1846; Reid 1996). Likewise, the shell image of the *L. sitkana* specimen in Min et al. (2004)’s encyclopedia is very similar to an abraded form of our *L. brevicula* specimens collected from Modong-ri (Figure 1B), which yielded a mtDNA *cox1* sequence that was not different from the typical shell morph of *L. brevicula* (Figure 1A) both in radula characters (Figure 2A, B) and mtDNA *cox1* sequence (Figure 3). Based on this morphological and molecular evidence, we consider that the *L. sitkana* in Min et al. (2004) is likely a misidentification of *L. brevicula*.

In addition, as a result of our morphological re-examination of Korean *Littorina* samples, we report a new record of *L. horikawai* from Jeju Island in Korea. The external shell ornamentation of *L. horikawai* and *L. sitkana* is similar, both having a shell surface with alternating dark brown and whitish bands and coarsely ribbed spiral ribs (but showing varying degrees of ecotypic variation). However, these two species differ in shell morphs: the body whorl of *L. horikawai* is relatively narrow with a tall, pointed spire, whereas *L. sitkana* has a well-inflated, rounded whorl with a low, blunt-ended spire. Despite their similarities in external shell characters, the mtDNA sequence of *L. horikawai* was 3% different from the *L. sitkana* sequence, and they were depicted as sister groups in phylogenetic trees (Figure 3). This is consistent with earlier phylogenetic analyses based on morphological (Reid et al. 1996) and/or molecular data (Reid et al. 2012).

In conclusion, we revisited the taxonomy and previous records of *Littorina* species reported from Korean waters using morphological (shell and radula) characters and *cox1* sequences. Our results support the revision of the Korean *Littorina* species list to comprise *L. brevicula*, *L. sitkana* and *L. horikawai,* and to exclude *L. kasatka*, which was previously erroneously reported from Korean waters.
